# Heading in the Right Direction: Understanding Cellular Orientation Responses to Complex Biophysical Environments

**DOI:** 10.1007/s12195-015-0422-7

**Published:** 2015-11-02

**Authors:** Chiara Tamiello, Antonetta B. C. Buskermolen, Frank P. T. Baaijens, Jos L. V. Broers, Carlijn V. C. Bouten

**Affiliations:** Department of Biomedical Engineering, Eindhoven University of Technology, P.O. Box 513, 5600 MB Eindhoven, The Netherlands; Institute for Complex Molecular Systems, Eindhoven University of Technology, P.O. Box 513, 5600 MB Eindhoven, The Netherlands; Department of Molecular Cell Biology, CARIM School for Cardiovascular Diseases, Maastricht University, P.O. Box 616, 6200 MD Maastricht, The Netherlands

**Keywords:** Mechanotransduction, Actin cytoskeleton, Focal adhesion, Strain avoidance, Contact guidance, Structural pathway

## Abstract

The aim of cardiovascular regeneration is to mimic the biological and mechanical functioning of tissues. For this it is crucial to recapitulate the *in vivo* cellular organization, which is the result of controlled cellular orientation. Cellular orientation response stems from the interaction between the cell and its complex biophysical environment. Environmental biophysical cues are continuously detected and transduced to the nucleus through entwined mechanotransduction pathways. Next to the biochemical cascades invoked by the mechanical stimuli, the structural mechanotransduction pathway made of focal adhesions and the actin cytoskeleton can quickly transduce the biophysical signals directly to the nucleus. Observations linking cellular orientation response to biophysical cues have pointed out that the anisotropy and cyclic straining of the substrate influence cellular orientation. Yet, little is known about the mechanisms governing cellular orientation responses in case of cues applied separately and in combination. This review provides the state-of-the-art knowledge on the structural mechanotransduction pathway of adhesive cells, followed by an overview of the current understanding of cellular orientation responses to substrate anisotropy and uniaxial cyclic strain. Finally, we argue that comprehensive understanding of cellular orientation in complex biophysical environments requires systematic approaches based on the dissection of (sub)cellular responses to the individual cues composing the biophysical niche.

## Introduction

Cardiovascular regenerative medicine has emerged as a promising approach to replace or regenerate damaged or diseased cardiovascular tissues. This interdisciplinary field, at the cross-section of engineering and life sciences, has the potential to restore normal cardiovascular function by using (the properties of) living cells in combination with biomaterials, genes, or drugs. Novel *in situ* tissue engineering approaches rely on the regenerative potential of the body itself by guiding and controlling cell behavior inside the human body with tailored biomaterials.

The premise of this approach is that, to recapitulate tissue function, an in-depth understanding of native cell behavior under physiological conditions and in response to a biomaterial is needed. Only then, strategies for controlling cell behavior can be designed towards the restoration of tissue functionality and mechanical integrity.^52^

One crucial, but often overlooked, aspect of mimicking native tissue functioning is obtaining and retaining cellular organization. The importance of cellular organization is demonstrated by the fact that biological and mechanical functioning of most tissues is dictated by the cellular arrangement.^42^

The tissues of the cardiovascular system are highly organized. For instance, the myocardial wall,^118^ heart valves^120^ and larger arteries^134^ are characterized by a layered structure with a well-defined cellular arrangement conferring the tissues their native unique anisotropic mechanical behavior needed to perform their function. Given the correlation between structural organization and function, it becomes clear that the loss of cellular organization is indicative of tissue malfunctioning, which can eventually lead to pathophysiological conditions. The disorganized arrangement of cardiac cells, for example, is a histological hallmark of cardiac dysfunction in hypertrophic cardiomyopathy.^23^,^58^,^61^,^102^

Cellular organization in cardiovascular tissues depends on the complex interactions between cells, the properties of the microenvironment and the cyclic strains resulting from the hemodynamic environment. Living adherent cells actively interact, respond, and adapt to biochemical and biophysical perturbations. These perturbations trigger intracellular signaling events leading to specific cellular mechanoresponses capable of directing biological relevant processes such as cell differentiation, proliferation and contractility. The mechanisms employed by cells to respond and adapt to the biochemical and biophysical cues of the micro-environment consist of a myriad of distinct but interconnected pathways whose details remain to be unraveled. The outside-in and inside-out feedback loop, referred to as mechanotransduction, is traditionally regarded as the process of converting mechanical stimuli into biochemical signals. Recently, it has been suggested that the structural pathway connecting the extracellular environment to the nucleus,^149^ here defined as “the structural mechanotransduction pathway”, might be as important as the biochemical transduction pathway for conducting biophysical signal to the nuclear interior. This new concept is supported by the fact that the long-range force propagation into the cell, resulting in deformations deep inside the cytoskeleton and nucleus, occurs 40 times faster than biochemical signaling.^97^ The structural mechanotransduction pathway consists of structural load bearing elements, such as integrins and focal adhesion complexes at the cellular membrane, and actin cytoskeleton stress fibres connected to the nucleus *via* so-called LINC (Linkers of the Nucleoskeleton and Cytoskeleton) complexes. Experimental evidence for this direct interconnection arises from studies where forces were applied directly to a small spot on the cell surface and consequently induced deformations and movements in the cellular interior.^91^,^93^ Clearly, defects in the complex and delicate interplay between the cell and its micro-environment resulting, for instance, from aberrations of the structural mechanotransduction pathway, may result in altered cellular mechanoresponse, in case no compensatory signaling mechanisms arise.

The recent development of micro-fabricated devices capable of effectively mimicking controlled biophysical cues has triggered numerous studies aiming at unraveling cellular responses to the properties of the micro-environment. It has become clear that cell orientation is actively determined by the actin stress fibres.^132^ Stress fiber orientation and, consequently, cellular alignment can be induced by two important biophysical cues of the cellular environment, such as those occurring during hemodynamic loading: (1) the anisotropy of the environment, e.g., the substrate on which cells are cultured and (2) uniaxial cyclic strain.^7^,^88^ These cues induce rapid and specific orientation of the intracellular elements of the structural mechanotransduction pathway, i.e., the focal adhesions, the actin cytoskeleton and the nucleus, suggesting that the direct structural mechanotransduction pathway plays a fundamental role in the cellular orientation response.^30^,^73^

Although a wealth of information has been obtained by recent *in vitro* mechanotransduction studies at the tissue-level, single cell observations provide detailed mechanistic insights towards a comprehensive understanding of cellular mechanotransduction. Yet, integrating the results of different investigations is a difficult task because of the complexity of the cellular response, which is not only highly dependent on the choice of the physical and mechanical experimental parameters, but also dependent on the cell-type. Moreover, the effects of combined biophysical cues on the cellular orientation response have just begun to be explored.

Here, we present a state-of-the-art review on the complex interplay between cells, topographical and cyclic strains cues of the extracellular environment, with a focus on cells of the cardiovascular system. Focusing on single cell observations, we first introduce the structural mechanotransduction pathway, i.e., the connected cellular components forming the physical link between the extracellular environment and the nuclear genome. Then, we continue our discussion with a review of experimental observations regarding cellular orientation response to anisotropy of the substrate and uniaxial cyclic strain in two-dimensional (2D) environments. We conclude with a brief outlook on future research directions for improving our current knowledge of cellular mechanoresponse to complex biophysical environments.

## The Structural Mechanotransduction Pathway: A Physical Connection Between the Extracellular Environment and the Genome

In this section we provide background information on the cellular structural components forming the structural mechanotransduction pathway, i.e., the physical connection between the extracellular matrix (ECM) and the genome contained by the nucleus.

**Figure 1:**
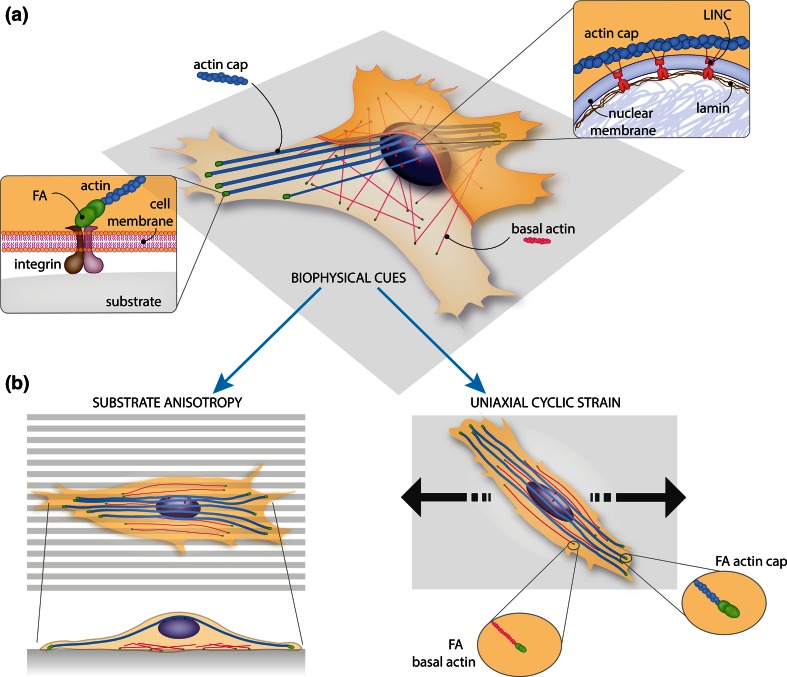
The structural mechanotransduction pathway and cellular orientation response to anisotropy of the substrate and uniaxial cyclic strain. (a) Schematic illustration highlighting the (protein) structural elements forming the structural mechanotransduction pathway. Integrins at the plasma membrane connect the extracellular environment (substrate) to the actin cytoskeleton. The connection is realized, in the cellular interior, by the focal adhesion complex. Within the actin cytoskeleton filaments, two kinds of fibers can be distinguished. The basal actin fibers (pink) that can be found underneath the nucleus and the actin cap fibers running on top of the nucleus (cyan). Actin cap fibers are connected to the nuclear interior *via* the LINC complex and lamins, a group of proteins underlying the nuclear membrane. This network of components forms a direct connection between the extracellular environment and the nuclear interior and function as a fast passing system for the biophysical stimuli. (b) Schematic illustration of cellular response to substrate anisotropy and uniaxial cyclic strain. When plated on an anisotropic substrate (left), the cell tends to align in the direction of the anisotropy. Focal adhesions as well as the actin cytoskeleton align accordingly. The side view shows the arrangements of the actin cap and basal actin fibers. Upon uniaxial cyclic strain (right), the cell responds by strain avoidance. The focal adhesions and the actin cytoskeleton align at an angle with respect to the straining direction. Overall cell orientation coincides with the actin cytoskeleton orientation. Note that the focal adhesions associated with the actin cap fibers are bigger than those associated with the basal actin fibers. Figure by Anthal Smits.

The structural components are represented by the focal adhesions situated at the cell membrane, the cytoskeletal filaments and, at last, the nucleus (Fig. 1). Among the cytoskeletal elements we concentrate on the actin filaments, since these structures are directly connected to the focal adhesions and play an important role in determining cell orientation.^131^,^154^ Moreover their behavior is relatively easy to analyze and quantify from microscopy imaging as they form anisotropic networks when cells are aligned.^10^,^154^ In this section also the relevance of the nucleo-cytoskeletal connections for correct mechanotransduction is elucidated.

### Interconnection Between the Extracellular Environment and the Actin Cytoskeleton

*In vivo* adhesive cells are embedded in a filamentous network called extracellular matrix (ECM). The integrins are the first components that physically link the ECM (outside of a cell) with the actin cytoskeleton (inside of the cell). Integrins are transmembrane αβ heterodimeric receptors that mediate cell adhesion to various ECM ligands such as collagen, fibronectin and laminin. The integrin family consists of about 25 members which are composed of combinations of α and β subunits, where the α subunit determines the ligand specificity for cell adhesion to the ECM.^68^ During cell adhesion, conformational changes in the integrins are induced by bidirectional (inside-out and outside-in) signaling of mechanical and biochemical signals across the cell membrane.^4^,^113^,^114^ Ligand binding to the integrins leads to clustering of integrin molecules at the cell membrane and recruitment of actin filaments inside the cell. The result of this process is the formation of the so-called nascent focal adhesion complexes (Fig. 1a, left inset), multi-molecular complexes that consist of a large number of different proteins, including talin, vinculin, paxillin and tensin.

Focal adhesion complex formation initially starts with immature, small structures (approximately 100 nm in diameter^45^). These structures reside at the leading edge in protrusions of the cells and provide the structural links between the ECM and the actin cytoskeleton. Strikingly, the maturation of the small focal adhesion complexes into bigger, mature focal adhesions is dependent on actin cytoskeleton bundling and generation of mechanical force. The actin cytoskeleton spans the whole cytoplasm of eukaryotic cells, continuously remodels and reorganizes to perform specific cellular functions.^95^,^140^ It is made of globular actin (G-actin), which continuously polymerizes into semi-flexible actin filaments, the filamentous actin (F-actin). F-actin assembles into bundles of fibres interconnected by actin crosslinkers (such as alpha-actinin and filamin) and motor proteins such as myosin II.^103^ These bundles of F-actin fibers are referred to as stress fibers. The presence of myosin II within the stress fibers is responsible for their contractility. The newly formed focal adhesions (FAs) reside in both central and peripheral regions of the cell. During this process the morphology of the FAs changes from a dot-like structure to a bigger and more elongated structure (2–10 *μ*m).^22^,^44^ This happens also as a consequence of the recruitment at the adhesion complex of several other proteins, for instance zyxin and alpha-actinin.^163^ A critical molecule for both maturation of FAs and mechanosensing is focal adhesion kinase (FAK). This molecule is involved in the transmission of external signals to the cytoskeleton by phosphorylation.^50^

The maturation of FAs provides stable adhesive interconnections between the stress fibers and the ECM. This allows the cell to probe its complex biophysical environment in various directions and over large temporal and spatial scales.^121^ Focal adhesions do not actively generate forces, but rather serve to regulate force transmission between the cytoskeleton and ECM.^104^ The actin cytoskeleton is the intracellular structure able to impose increasing forces when facing growing resistance. This confers the actin cytoskeleton intrinsic mechanosensing and ability to adapt to developing mechanical cues of the cellular environment. However, to which extent stress fibers participate in sensing and transducing environmental signals has not been fully elucidated yet.

#### Diseases Associated with Dysfunctional ECM-Actin Connections

The relevance of correct functioning of all components within the mechanotransduction route between ECM and the actin cytoskeleton has become clear in numerous studies in the past few years.

Starting at the ECM, its composition appears to have major impact on cellular behavior. Either weakening or increased ECM stiffness due to decreased or increased amount of collagen evokes a cellular response, which, upon disturbed mechanotransduction, can lead to an even more deregulated ECM. For example, cardiac tissue damage that normally causes controlled levels of enhanced myofibroblast proliferation and collagen production, will, without proper feedback due to disturbed mechanosensing, lead to cardiac fibrosis and stiffening of the cardiac muscle.^92^ For a recent review on the interplay between ECM and mechanotransduction, see Ref. 43

Not only amounts of ECM components but also abnormalities in the molecular composition of its components lead to disturbed mechanosignaling. For instance, mutations in the ECM protein fibrillin-1 as seen in the Marfan syndrome, cause the development of cardiomyopathies in affected mice due to disturbed mechanosignaling. Normalizing the extracellular matrix composition in these mice resulted in disappearance of these symptoms.^25^

Proteins, assembled in the FA complex, including the transmembrane integrins as well as the other binding partners, mostly localized at the cytoplasmic side of the cellular membrane, all appear to be critical for the transmission of extracellular forces. Especially, any structural abnormalities in integrins have a devastating effect on mechanotransduction. Even minor modifications of the β1-integrin gene resulted in multiple defects in mechanotransduction signaling in adult cardiomyocytes.^86^ An overwhelming number of diseases now have been assigned to failure in any of the other components of the FAs, based on mouse models.^90^ While not all of these diseases are caused by disturbed mechanotransduction, it has become clear that especially improper functioning of the key enzyme FAK leads to severe abnormalities in heart development and heart failure.^117^

While this brief overview suggests that most mechanotransduction diseases are associated with heart (muscle) tissue there is growing evidence that impaired or sustained mechanotransduction at the cellular boundaries leads to several other diseases. Abnormal mechanical stimulation can switch on signaling pathways such as beta-catenin signaling in adenomatous polyposis coli (APC) deficient colon tissue, stimulating the development of colon cancer.^156^ Moreover ECM stiffness can drive epithelial to mesenchymal transition of cancer cells,^153^ increasing the malignant behavior of these cells.

### Interconnection Between the Actin Cytoskeleton and the Nucleus

In the surrounding of the nucleus, a subset of actin stress fibers have been found to organize in thick parallel and well-ordered bundles of fibers, physically anchored to the apical surface of the nucleus.^75^,^76^ Wirtz and co-workers have made an effort to characterize these fibers (actin cap) which are strikingly terminated by wide, long and dynamic focal adhesions (Fig. 1a).^17^,^64^,^75^ First, they have demonstrated that the actin cap stress fibers differ from the conventional stress fibers found below the nucleus (basal actin layer, Fig. 1b). By containing more myosin II and the actin bounding protein alpha-actinin, actin cap stress fibers are very contractile and highly dynamic.^98^ Furthermore, these fibers not only play a major role in shaping and positioning the nucleus,^19^,^64^,^75^,^77^,^98^,^139^ but they are also involved in mechanosensing of substrate elasticity. For instance, cells without an actin cap were observed to be less responsive to changes in matrix elasticity. Finally, fast mechanotransduction also seems to be enabled by this subset of stress fibers. In their study, Chambliss *et al.* showed that, in response to shear stress stimulation, cells without the actin cap build up thick stress fibers in a shorter time span as compared to in response to biochemical stimulation.^18^ From these findings it has become clear that the perinuclear actin cap is a key component of the physical pathway from the ECM to the nuclear interior for mechanosensing and mechanotransduction.

The coupling between the perinuclear actin cap and the nucleus (nucleo-cytoskeletal connection) is mediated by a group of recently discovered proteins, referred to as the LINC complex (Linker of Nucleoskeleton and Cytoskeleton, Fig. 1a right inset).^26^,^108^,^127^ Hooking at the cytoplasmic side of the nucleus, on the outer nuclear membrane (ONM), we find the nesprins (KASH domains proteins), which are connected to the various cytoskeletal filaments.^125^,^166^ Among the four variants of nesprins, nesprin-1 and -2 bind to actin filaments.^54^ Nesprins, in turn, bind to SUN domain proteins spanning the whole nuclear envelope reaching the nuclear interior. SUN proteins then bind to lamins, a family of type V intermediate filaments underlying the inner nuclear membrane (INM).^56^ Lamins in turn physically connect to chromatin. Thus, in this way a physical bridge is formed from the cellular exterior *via* focal adhesion complexes, actin, the LINC complex, and lamins to chromatin.

Lamins form an elastic meshwork called nuclear lamina (Fig. 1a, right inset).^29^,^109^ Lamins consist of two main subtypes, A- and B-type lamins (encoded by the gene LMNA, or LMNB1 and LMNB2 respectively).^53^ While B-type lamins are essential for cell survival, A-type lamins are thought to contribute significantly to the maintenance of mechanical integrity of the nucleus.^12^,^63^,^80^,^138^ The nuclear lamina interacts also with the chromatin of the nucleoplasm, and therefore plays a major role in gene expression, DNA replication and repair, chromatin organization and transcriptional response.^35^,^49^,^122^,^168^

The role of the nucleo-cytoskeletal connection in force transmission has been examined recently by many groups. The results of various experimental approaches based on two- and three-dimensional substrates or application of mechanical load, have shown that the structural integrity of this connection is indeed needed for propagation of forces to the nucleus. Indirect demonstration has come from studies employing LMNA-depleted cells. By using this model, it has been shown that nuclear deformations in response to local cellular membrane stretch are completely abolished.^91^ In addition, the studies by Poh *et al.*^111^ and Zweger *et al.*^169^ have provided direct evidence that forces are not transmitted to the nucleus when LMNA is depleted from cells, thus when the nucleo-cytoskeletal connection is lost. Recently, it has emerged as well that the tension exerted by the actin on the nucleus directly mediates the spatial polarization of nuclear lamina and the intranuclear architecture.^78^ In cells lacking A-type lamins, the formation of a nuclear actin cap is partially abolished.^75^ Also, the impaired activation of mechanosensitive genes has been reported in studies with cells lacking A-type lamins.^62^,^81^

A number of other studies in which either the LINC complex was disrupted or a loss of lamins was induced, support these findings adding that also other cellular functions such as migration, polarization and developmental processes become affected.^14^,^83^

Although the role of the LINC complex in force propagation to the nucleus has been clarified, controversy remains about its impact on the activation of mechanotransduction pathways. Clues to understand these mechanisms might come from studying diseases arsing form mutations in any of the components connecting actin to the nucleus.

#### Diseases Associated with Defective Actin-Nucleus Coupling

Mutations in the LMNA gene encoding for A-type lamins in the nuclear lamina cause a broad spectrum of genetic diseases, collectively referred to as laminopathies.^13^ Several hundred mutations in the gene have been discovered and most of them have tissue-specific phenotypes. Twelve different diseases are included into this group: those affecting striated muscle (ranging from Emery/Dreifuss muscular dystrophy (EDMD) to dilated cardiomiopathy with conduction system defects (DCM-CD) and Limb-girdle muscular dystrophy (LGDM), those affecting the adipose tissue (partial lipodystrophy of Dunningan type (FPLD) and those affecting the nervous system (Autosomal recessive Charcot-Marie-Tooth type 2 and Autosomal dominant axonal Charcot-Marie-Tooth disease). However, primary laminopathies can also affect tissues in a systemic fashion and cause premature-aging syndromes like Restrictive Dermopathy (RD) and Hutchinson-Gilford progeria syndrome (HGPS).^159^ The mechanisms underlying tissue-specific effects observed in laminopathies are still largely unknown. Especially in the most diffuse laminopathies, the muscular dystrophies and cardiomyopathies,^16^ it might well be that the lack of structural integrity, thus the susceptibility to mechanical stress could result in altered chromatin organization which, on its turn, results in altered gene expression.

Recently it was observed that also mutations in other components of the LINC complex (e.g., emerin, nesprin-1, nesprin-2, etc.) can give rise to the same disease pathology as seen in EDMD due to LMNA mutations.^8^,^87^,^94^,^164^ Next to this, combinations of mutations in the nucleo-cytoskeletal system have been shown to lead to more severe diseases than the individual component mutations.^85^,^96^,^129^ At the same time, it has been suggested that the biochemical signals coming from the cytoplasm might take over or compensate for the lack of the physical nucleo-cytoskeletal interconnection.^14^,^91^

Altogether, the examples above demonstrate that several structural components of the mechanotransduction pathway connecting the cellular micro-environment and the nuclear interior have been identified. While we do not know the degree of completeness of our understanding, we can confidently state that the structural interconnection is crucial for determining the cellular mechanoresponse.

## Cellular Orientation Response to Substrate Anisotropy and Cyclic Strain

In the previous section, in order to appreciate the inside-in part of the cellular mechanotransduction, i.e., how environmental signals are transmitted to the nucleus, we introduced the components of the structural mechanotransduction pathway interconnecting the extracellular environment and the nucleus. To get a comprehensive understanding of the interplay between cellular responses and complex biophysical environments, it is also necessary to have a deep understanding of the inside-out signaling used for cellular mechanoresponse, i.e., how cells respond to environmental cues and which mechanisms are employed by cells for mechanoresponse. In this section we discuss the cellular orientation response to substrate anisotropy and uniaxial cyclic strain (Fig. 1b), focussing on the main components of the structural mechanotransduction pathway, i.e., the focal adhesions, the actin cytoskeleton and the nucleus.

### Cellular Orientation Response to Substrate Anisotropy

Various biophysical cues such as topography, cyclic strain and the mechanical properties of the extracellular environment can induce the alignment of adherent cells by promoting an anisotropic arrangement of structural components at the subcellular level. In 1912, Harrison reported for the first time that the topography of a substrate could influence cell behavior.^57^ Weiss confirmed this in 1945, with the observation that cells preferentially orient and migrate along fibers, an organization principle he named contact guidance.^155^ Today the connotation of this term is slightly different. Contact guidance is now regarded as the ability of cells to sense and align with the anisotropy of the surrounding micro-environment.

Recent developments in microfabrication technologies have led to the manufacturing and application of a variety of substrates with different geometries and length scales, from which several substrates can be used to study contact guidance. Observations obtained using microfabricated substrates engineered to induce contact guidance, have confirmed that a variety of tissue cells, ranging from endothelial cells,^38^,^135^,^136^ to fibroblasts,^37^,^39^,^105^,^141^,^142^ and smooth muscle cells^116^ orients along the direction of the anisotropy of the substrate. A summary of illustrative studies showing the response of cells of the cardiovascular system to anisotropic features of the culture substrate in the sub-micrometer to micrometer scale is reported in Table 1.

**Table 1:** Experimental investigations on cell orientation response induced by anisotropy of the substrate.

Cell type	Method	Parameters	Main observed results	Source
Fibroblasts (REF52 cells)	Parallel microcontact printed fibronectin lines Glass	Lines: 2 *μ*m wide separated by 4, 6, 10 or 12 *μ*m wide stripes	Focal adhesions are formed at the adhesive lines If spacing is larger than 6 *μ*m, focal adhesions orient either perpendicular to the lines or orient in the direction of the lines Actin stress fibres can cross several stripes when the non-adhesive spacing is smaller than 6 *μ*m With wider spacing stress fibres form between adjacent adhesive stripes or along single stripes	Zimerman *et al.* 167
Chick heart fibroblasts	Parallel grooves Quartz	Ridge width from 1.65 to 8.96 *μ*m Groove width from 3 to 32 *μ*m Constant depth: 0.69 *μ*m	Focal adhesions observed on the floor of the grooves and at the ridges Actin bundles associated with focal contact on the floor of grooves are parallel to the groove axis and hardly ever nearly perpendicular to this axis. No such restriction is found on the ridges	Dunn and Brown^39^
Human gingival fibroblasts	Parallel grooves Silicon Titanium coating	Ridge and groove width of 15 *μ*m Constant depth: 3 *μ*m	Microtubules located at the bottom of the grooves are the first component to align along the grooves. Subsequently, focal adhesions and actin microfilaments align At a single groove or ridge, focal adhesions are oriented both parallel and perpendicular to the groove direction	Oakley and Brunette^105^
Rat dermal fibroblasts	Parallel grooves PDMS RFGD treatment	Ridge and groove width from 1 to 10 *μ*m Depth: 0.45 or 1 *μ*m	Cells at surfaces with a ridge width smaller than 10 *μ*m elongate along the surface grooves. If the ridge width is larger than 4 *μ*m, cellular orientation was random and the shape of the cells became more circular. The ridge width is the most important parameter, since varying the groove width and groove depth does not affect cell size, shape, nor the angle of cellular orientation	den Braber *et al.* 37
		Ridge and groove width from 1 to 20 *μ*m Depth: 0.5, 1, 1.5, 1.8, 5.4 *μ*m	The cells always elongate in the groove direction without any significant difference in behavior between a 2–20 *μ*m wide grooves. However, groove depth affects the cellular orientation	Walboomers *et al.* 142
		Ridge and groove width from 1 to 10 *μ*m Constant depth: 0.5 *μ*m	Actin fibers orient in direction of the grooves. This happens more rapidly on the narrow grooves The addition of cytochalasin-B only causes delay in cell attachment and spreading, thus a well-formed cellular actin cytoskeleton is no prerequisite for the occurrence of contact guidance	Walboomers *et al.* 141
Myofibroblasts (HVSCs)	Elastomeric microposts PDMS microcontact printed Human Plasma Fibronectin on top of microposts	Elliptical microposts: *a* = 1.5 *μ*m, *b* = 0.87 *μ*m Circular microposts: *d* = 2 *μ*m	Elliptical microposts: orientation in the direction of the major axis of the ellipse, even for very stiff microposts. Topographical cues induce cellular alignment	Tamiello *et al.* 128
Vascular smooth muscle cells	Parallel grooves PDMS Fibronectin coating	Ridge width: 12 µm Groove width: 20, 50, and 80 µm Constant depth: 5 µm	For all groove widths investigated, cells align in the direction of the microgrooves The actin filaments are highly aligned and parallel to the grooves on the smallest groove widths As the groove width increases to 50 and 80 µm, there is a clear decrease in the number of highly aligned fibers	Sarkar *et al.* 119
	Parallel microcontact printed fibronectin or laminin lines PDMS	Lines width from 20 to 100 *μ*m, separated by 100 *μ*m wide stripes	Actin cytoskeleton aligns along all patterns	Alford *et al.* 3
Aortic smooth muscle cells	Parallel grooves PDMS Gelatine, fibronectin, vitronectin or poly-d-lysine coating	A PDMS sheet was stretched uniaxially using a custom-made stretcher to produce a fixed amount of prestretch The stretched sheet was treated with oxygen plasma for a fixed period of time. This resulted in substrates with or without parallel grooves.	Focal adhesions are more prone to become mature when they run along microgrooves, causing mature focal adhesions to align in the direction of the microgrooves. These adhesions have straighter actin bundles oriented parallel to the microgrooves	Saito *et al.* 116
Bovine aortic endothelial cells	Parallel grooves PDMS Fibronectin coating	Ridge width from 3 to 5.5 *μ*m Groove width from 3 to 4 *μ*m Depth: 200 nm, 500 nm, 1 *μ*m, 5 *μ*m	Majority of the focal adhesions and actin fibers orient in direction of the ridges Focal adhesions localized at the ridge edges and along the sidewalls of 1 *μ*m deep microgrooves No focal adhesions on the bottom of the 5 *μ*m deep microgrooves	Uttayarat *et al.* 135
Endothelial cells (HUVECs)	Parallel grooves Silicone Human Plasma Fibronectin coating	Ridge and groove width of 2, 5, and 10 *μ*m Constant depth: 5 *μ*m	On 2 *μ*m surfaces the vast majority of focal contacts are deposited on the ridges On the 5 *μ*m and 10 *μ*m surfaces, cells are increasingly able to form these contacts also in the grooves	van Kooten and von Recum^136^
	Parallel grooves PDMS Serum-free medium and without protein coating	Ridge and groove width from 200 to 2000 nm Constant depth: 300 nm	HUVECs orient parallel to the long axis of underlying ridges, even in the absence of added protein The actin stress fibers align parallel to the ridges and grooves on patterned substrates	Dreier *et al.* 38
Epithelial (MDCK) cells	Elastomeric microposts PDMS Microcontact printed fibronectin on top of microposts and microcontact printed fibronectin elliptical islands glass	Elliptical microposts: *a* = 0.95 *μ*m, *b* = 0.55 *μ*m Elliptical adhesive islands: *a* = 0.95 *μ*m, *b* = 0.55 *μ*m	Elliptical posts: orientation of the focal adhesions and actin cytoskeleton in the direction of the major axis of the ellipse Elliptical islands: no preferential orientation of the focal adhesions and actin cytoskeleton. Alignment is induced by differential stiffness of elliptical microposts	Saez *et al.* 115

*HUVECs* human umbilical vein endothelial cells, *MDCK* Madin–Darby canine kidney, *PDMS* polydimethylsiloxane, *RFGD* radio frequency glow discharge

**Figure 2:**
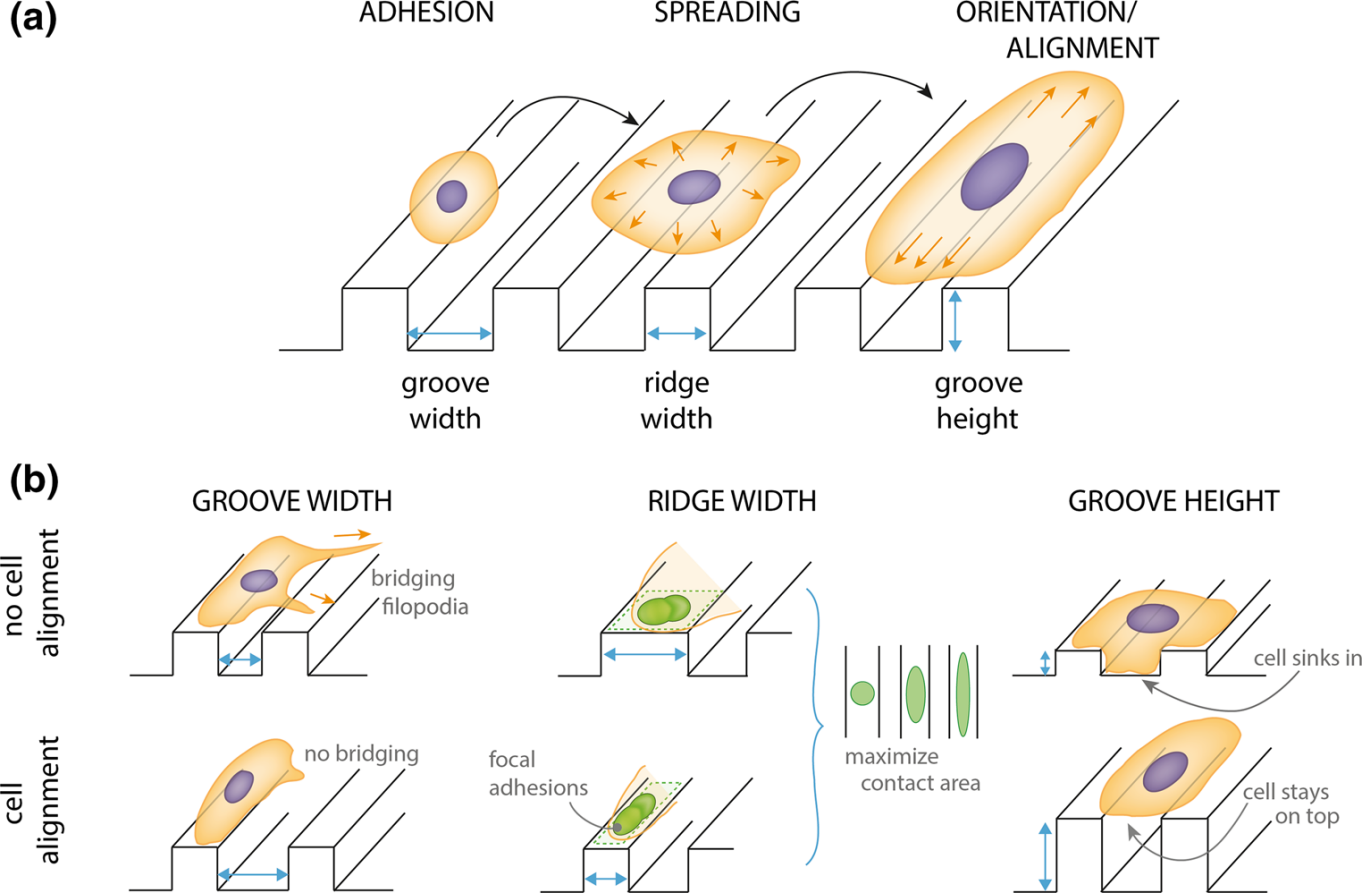
Cellular orientation response to microgrooves. (a) Schematic illustration showing the overall cellular orientation response from cell adhesion to alignment on a microgrooved substrate. At the moment the cell adheres to the microgrooved substrate, the cell undergoes spreading followed by cell alignment, i.e., orientation along the direction of the microgrooves, a phenomenon called contact guidance. The parameters characterizing the microgrooved substrate are pointed out with light blue arrows: groove width, ridge width and groove height. (b) Schematic representation of the proposed mechanisms explaining contact guidance in relation to the microgroove’s parameters. (Top) No cell alignment and (bottom) cell alignment. (Left) groove width—mechanical restriction theory. When the microgrooves are too narrow, cell’s filopodia succeed in bridging the space between two consecutives ridges. Therefore, the cell does not align (top). When the width of the microgrooves increases, filopodia are not able to bridge two consecutive ridges, giving the signal for cell alignment in the direction of the microgrooves (bottom). (Center) ridge width—focal adhesion theory. Ridge width influences the orientation and maturation of focal adhesions. Wide ridges do not impose geometrical confinement on the focal adhesion (green). Therefore, the maturation of the focal adhesions can occur in both directions, preventing any cell alignment (top). Narrower ridges impose geometrical confinement to the focal adhesions, which tend to maximize their contact area with the substrate. As a result, focal adhesions align and mature in the direction of the ridges, i.e., the direction of the microgrooves (bottom). (Right) groove height—discontinuity theory. For low microgrooves, the cell sinks into the microgrooves and, consequently, it does not align in direction of the microgrooves (top). For sufficiently high microgrooves, the cell senses the discontinuities of the microgrooves represented by their edges and forms focal adhesions only on the ridges. Consequently, the cell aligns in the direction of the microgrooves (bottom). Figure by Anthal Smits.

The most used substrates for studying contact guidance are microgrooves, i.e., microengineered arrays of parallel micrometer-sized grooves and ridges. When culturing adherent cells on these substrates it is observed that, at the subcellular level, the focal adhesions and actin fibers follow cellular orientation (Fig. 1b, left). However, the specific response of these structural cellular components depends on many parameters such as groove width,^38^,^39^,^119^,^136^,^142^ ridge width,^11^,^37^–^39^,^130^,^136^ groove height^11^,^24^,^130^,^135^,^142^ and surface treatment.^38^ The general trend is that when either the groove width or groove height increases, the cell forms focal adhesions on the ridges and consequently orients in their direction. Next to these observations, several theoretical frameworks have been elaborated for explaining cell alignment in relation to the microgroove’s parameters. The schematic representation of these theories is shown in Fig. 2.

- T**he mechanical restriction theory** by Dunn and Heath focuses on the relative inflexibility of cytoskeletal structures as a primary regulator of cellular alignment.^40^ The shape of the substratum is demonstrated to impose mechanical restrictions for the formation of cytoskeletal protrusions, called filopodia, as recently shown also by Zimerman *et al.*^167^ and Ventre *et al.*^137^. According to this theory, the distance between the anisotropic features, either the **groove width** (Fig. 2b, left), on microgrooved substrates or the distance between adhesive lines on flat substrates, is the crucial factor for cell alignment. If this distance cannot be bridged by the formation of any filopodia, cells become highly polarized and elongate in the direction of the substrate anisotropy. When cells align because of this mechanism, actin filaments as well as long focal adhesions are observed in the direction of the anisotropy. These focal adhesions are usually anchored to thick stress fibres and, therefore, are presumably the focal adhesions of the actin cap stress fibers.
- The **focal adhesion theory** by Ohara and Buck proposes that the orientation of cells is caused by the tendency of focal adhesions to maximize their contact area.^107^ According to this theory, on a microgrooved substrate, focal adhesion maturation and, consequently, cell alignment occur along the ridge only if **ridge width** (Fig. 2b, center) is comparable to the size of a focal adhesion. An argument against this theory is the observation of focal adhesions oriented both perpendicular and parallel to ridges of the microgrooved substrate.^105^,^137^,^167^ However, as pointed out by Ventre *et al.,*^137^ focal adhesions observed perpendicular to the ridge direction are unstable and connected to isolated actin fibers, while the focal adhesions parallel to the ridge are mature and connected to stress fibers. This ultimately guides cellular orientation in the ridge direction.
- **Discontinuity theory**: more recently Curtis and Clark proposed the idea that sharp discontinuities in the substrates, e.g., edges of microgrooves, induce cell alignment by triggering, first, actin condensations in these locations and, consequently, promoting focal adhesion formation at the same place.^27^ Despite the fact that this theory includes both the involvement of focal adhesions and actin filaments in cell alignment, it raises the question of how cells sense discontinuity, as already discussed by Curtis *et al.*^28^ Clark *et al.*^24^ observed that by increasing the **groove height** (Fig. 2b, right), more cells orient in the direction of the microgrooves. Based on these observations, it is proposed that for sufficiently high microgrooves, cells are more exposed to substrate discontinuity and, as a result, align along the microgrooves.

Although these theories have shed light on the possible mechanisms behind contact guidance, the role of each individual structural component has not been fully elucidated yet. A straightforward approach to investigate the influence of the actin cytoskeleton in cellular alignment to microgrooves consists by inhibiting the actin cytoskeleton *via* disrupting agents, such as performed by Walboomers *et al.*^141^ and Gerecht *et al.*^46^ On one hand, Walboomers *et al.* observed that fibroblasts can still align along the microgrooves even if the polymerization of actin is inhibited with the use of cytochalasin-B.^141^ Contrarily, Gerecht *et al.* found that by adding actin disrupting agents to human embryo stem cells on sub-micrometer sized grooves, the morphology of the cells gets rounder.^46^ These results illustrate that there is no consensus yet on the role played by the actin cytoskeleton in the cellular response to contact guidance.

**Figure 3:**
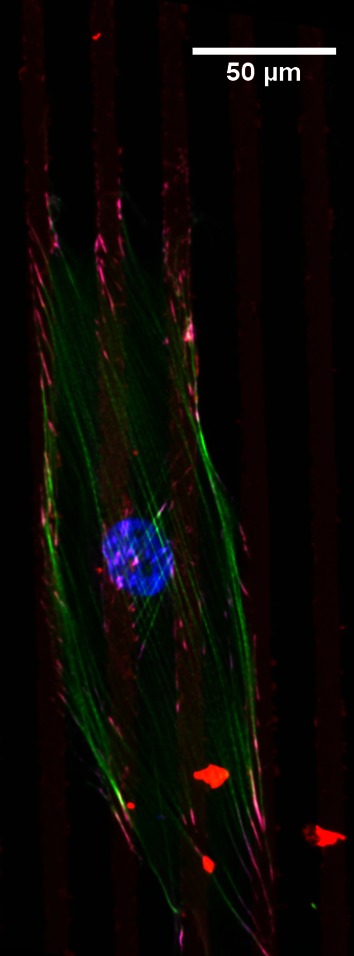
Cellular orientation response to a two-dimensional anisotropic environment. Representative microscopy image of a myofibroblast (Human Vena Saphena Cell) cultured on top of microcontact printed fibronectin (red) lines (10 *μ*m width and 10 *μ*m spacing) on polydimethylsiloxane (PDMS). The focal adhesions are stained in magenta, the actin stress fibers in green, and the nucleus in blue. The cell orients in direction of the lines. The focal adhesions and the actin stress fibers follow cellular orientation.

To unravel the relevance of each of the cellular components in the contact guidance phenomenon, a systematic approach is, in our view, needed. The various substrate features creating anisotropy need to be dissected (e.g., height, edges, biochemical patterning) and it is necessary to distinguish between substrate anisotropy by biochemical features (e.g., geometrical features given by printing of extracellular matrix proteins), i.e., a purely two-dimensional (2D) environment, and substrate anisotropy by topographical features (e.g., pillars, posts, microgrooves, fibers), here named two-and-a-half-dimensional (2.5D) environment. The first step towards this systematic approach, is neglecting the influence of the height of topographic features (e.g., discontinuity). Thus, as a first step, it is suggested to study contact guidance in 2D environments. Pure biochemical anisotropic features can be produced for instance by microcontact printing. According to this methodology, an elastomeric stamp of polydimethylsiloxane (PDMS) incubated with an extracellular matrix protein (e.g., fibronectin) can be used to create adhesive patterns on flat surfaces, such as glass or PDMS. The bare regions are then backfilled with a non-adhesive protein or polymer, to avoid non-specific cell adhesion. Microcontact printing has proven to be a useful technique to adhere cells to single or multiple islands.^20^,^21^ In this way one can geometrically control cell adhesion to regulate cell functions. However, there are only limited studies where this technique has been used to induce cellular alignment *via* printed lines whose width is in the order of micrometers.^3^,^158^,^167^ In our view, this kind of studies will elucidate the precise mechanisms behind cellular alignment (Fig. 3).

### Cellular Orientation Response to Uniaxial Cyclic Strain

**Figure 4:**
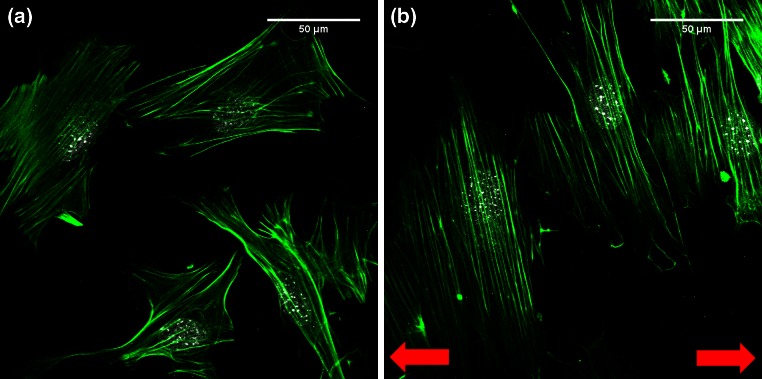
Cellular orientation response to uniaxial cyclic strain. (a) Mouse embryonic fibroblasts (MEFs) cultured in static conditions for 6 h on a homogenously fibronectin-coated silicone membrane and stained for actin stress fibers (green) and nucleus (white). Cells and actin stress fibers are oriented randomly. (b) MEFs after 24 h of uniaxial cyclic strain (7%, 0.5 Hz). Cells and stress fiber are oriented almost perpendicularly to the strain direction (red arrows). This response is called strain avoidance.

Cellular response to uniaxial cyclic strain is demonstrated by the dynamic reorganization and reorientation of cells and stress fibers. It has become clear that stress fibers play a crucial role in cell ability to remodel and respond appropriately to cyclic strain. Indeed, stress fiber disruption causes inhibition of cellular reorientation.^47^,^59^,^145^ In the 80s, the response of tissue cell to strain was for the first time observed and interpreted as an avoidance response to the strain of the substrate on which the cells were cultured, the so called strain avoidance response (Fig. 1b, right, Fig. 4).^15^ Since then, further studies have highlighted that, on 2D substrates, cell reorientation occurs at angles (nearly) perpendicular to the stretch direction, i.e., the direction of minimal substrate deformation (Fig. 4). In the last decades, several studies have been carried out in order to quantify and unravel the mechanisms of this phenomenon. Stretch avoidance appears to be a behavior belonging to many kinds of tissue cells, ranging from endothelial cells,^6^,^31^,^66^,^73^,^74^,^82^,^101^,^144^,^145^,^157^,^161^ to fibroblasts^9^,^70^,^100^ and smooth muscle cells.^32^,^71^,^126^ However, the dependence of such response on the spatiotemporal parameters of the cyclic stimulation, such as frequency,^66^,^70^,^88^ magnitude,^9^,^32^,^66^,^71^,^73^,^144^,^157^ strain rate,^67^,^82^,^100^,^133^ duration,^160^,^161^,^165^ or even the combination of some of those,^148^ makes any attempt to correlate the effects of these factors with cellular response unsuccessful. Moreover stretch avoidance response seems to be cell type dependent and a minimal strain amplitude,^6^,^32^,^100^ frequency^66^,^67^,^70^ or cell contractile status^41^ may be required for the response to occur. Summary of studies about cells of the cardiovascular system (fibroblasts, tissue cells, endothelial and progenitor cells) and stress fiber response to uniaxial cyclic stretch are reported in Table 2.

**Table 2:** Experimental investigations on cell and stress fiber orientation response upon uniaxial cyclic strain

Cell type	Method	Parameters	Main results	Source
Human Aortic Endothelial Cells	Custom-built device Silicone membranes ProNectin-F coating^148^	10% at 0.5 Hz for 3 h	Reorientation is inhibited by stress fiber disruption	Wang *et al.* 145
		5 and 10% at 0.5 and 1 Hz for 3.5 h	Reorientation depends on stretch magnitude	Wang *et al.* 144
		5 and 10% at 0.5 to 2 Hz	Reorientation depends on stretch magnitude Cell reorientation is independent of SFs presence	Wille *et al.* 157
	Custom-built device Silicone membranes Fibronectin coating	10% at 10%/s for 3 h For time lapse: 5% at a constant rate of 5%/s for 6 h	Cytoskeletal reorganization within few seconds from onset of stretching SFs break and disassemble FAs loosen and cells become nearly round	Ngu *et al.* 101
Human Umbilical Vein Endothelial Cells	Custom-built device Silicon membranes Plasma treated	10% at 1 Hz for 3 h	Threshold for reorientation 1.8% strain magnitude	Barron *et al.* 6
	Custom-built device Silicon membranes Collagen type I coating	10% at 0.5 Hz for 0 to 20 h	The variance of actin fiber orientation became smaller after 2 h of stretch Actin fiber density increased within 30 min of stretching and decreased after 10 h of stretching	Yoshigi *et al.* 161
Gottinger Minipigs Aorta Endothelial Cells	Custom-built device Silicon membranes Collagen type I coating^32^	15% at 1 Hz for 3 days	Strain avoidance observed both in SFs and cells	Dartsch and Betz^31^
Bovine Aortic Endothelial Cells (BAECs)	Custom-built device Silicon membranes Fibronectin coating	10% at 1 Hz for 6 h	SFs reorientation depends on interplay between Rho pathway activity and the stretch magnitude	Kaunas *et al.* 73
		10% at 1 Hz for 8 h	Stretch-induced remodeling of the actin cytoskeleton modulates JNK signaling in response to cyclic stretch	Kaunas *et al.* 74
		0 to 20% at 0.01 to 1 Hz for 4 h	Reorientation depends on frequency (optimal at 1 Hz)	Hsu *et al.* 66
		10% at 0.01 to 1 Hz for 4 h	SF reorientation depends on strain rate Threshold 1 Hz for reorientation	Hsu *et al.* 67
BAECs and U2OS Osteosarcoma cells Stably expressing GFP-actin	Custom-built device Silicon membranes Fibronectin coating	10% at 0.1 and 1 Hz for 2 h	Reorientation depends on strain rate FAs and SFs distal ends do not disassemble SFs exhibit heterogeneous behavior within the cell, but also along the length of individual SFs	Lee *et al.* 82
Rabbit Aortic Smooth Muscle Cells	Custom-built device Silicon membranes Collagen type I coating	2 to 20% at 1 Hz for 3 to 12 h	Threshold 2% for reorientation Reorientation depends on stretch amplitude SF reorient prior to the reorientation of the cell bodies	Dartsch and Hammerle^32^
		2 to 10% at 1.2 Hz for 14 days	Reorientation depends on stretch amplitude	Dartsch *et al.* 33
a7r5 Rat Aortic Smooth Muscle Cells	FlexCell Collagen I-coated plates	100 to 124% of cell resting length at 1 Hz for 48 h	Cellular reorientation is independent of stretch-activated calcium channels Alignment is reversible after 48 h from stretch cessation Reorientation depends on stretch amplitude	Standley *et al.* 126
Rat Aortic Smooth Muscle Cells		10% at 0.5 to 2.0 Hz for 24 h	SFs are needed for reorientation 1.25 Hz, the most effective frequency for reorientation	Liu *et al.* 88
A10 Rat aortic Smooth Muscle Cells	Custom-built device Silicone membranes Collagen type I coating	20% at 1 Hz for 3 h	SFs reorient within 15 min after the onset of stretching Cells reorient within 1–3 h	Hayakawa *et al.* 59
		1.2 times cell original length at 1 Hz for 3 h	Cell orientation but not SF reorientation depends on stretch-activated calcium channels Rapid withdrawal of the cell periphery located in the direction of stretching and gradual extension toward the direction oblique to the stretching axis	Hayakawa *et al.* 60
*A10 Rat Aortic* *Smooth Muscle Cells* *Transfected EGFP-tagged moesin (fragments with actin-binding ability)*			*SFs aligned along the stretching direction are torn into pieces soon after stretching, and then reorient obliquely to the direction of stretching*	*Hayakawa* *et al.* 60
Earle’s Fibroblast	Custom-built device Silicon membrane	Elongation and recoil at 15 s intervals for 18 to 24 h	Pioneering study: Strain avoidance is observed	Buck^15^
Human Dermal Fibroblasts	Custom-built device Silicone membranes ProNectin-F coating	4 to 12% at 1 Hz for 24 h	Reorientation depends on combination of strain rate and amplitude Threshold for reorientation is 4.2% for fibroblasts	Neidlinger-Wilke *et al.* 100
		8% at 1 Hz for 24 h	Reorientation starts within 2-3 h from stretch onset and is complete at 24 h	Neidlinger-Wilke *et al.* 99
MRC5 Lung Human Fibroblasts	Instron 5564 testing Instrument Silicone sheets Fibronectin coating	1 to 25% at 0.5 Hz and 2% at 0.25 to 3 Hz for 3 h	Reorientation depends on strain amplitude Rapid response of cytoskeleton to strain	Boccafoschi *et al.* 9
Primary Human Umbilical Cord Fibroblasts	Custom-built device PDMS membranes of 1, 3, 11 and 50 kPa Fibronectin coating	4.9 to 32% at 9 to 52 mHz for 16 h	Threshold in amplitude On very soft substrates no reorientation occurs, even for high strain Changes in cell shape follow cytoskeletal reorientation with a significant temporal delay	Faust *et al.* 41
REF-52 Rat Embryonic Fibroblasts and Human Dermal Fibroblasts	Custom-built device Silicon membranes Fibronectin coating	1 to 15% at 0.0001 to 20 s^−1^ for 8 h	Reorientation depends on strain frequency (from 1 to 5 h)	Jungbauer *et al.* 70
*NIH3T3 Fibroblasts* *Transfected with GFP-Vinculin*	*Custom-built device* *Silicon membranes* *Fibronectin coating*	*8% at 1 Hz for 3 h*	*FAs reorient by sliding, without turnover and reassembly* *Reorientation is independent of microtubules but dependent of actin stress fiber presence*	*Goldyn * *et al.* 48
*Monkey Kidney Fibroblasts* *Transfected with GFP-Actin*	*Custom-built device* *PDMS membranes* *Fibronectin coating*	*16 and 28% for 3 h ca.*	*Reorientation occurs by dynamic rotation of intact actin stress fibers in fibroblasts* *Subcellular reorganization begins within minutes from strain application* *SFs at cell center region rotate, while SFs at cell periphery remain stable*	*Ahmed * *et al.* 1
*NIH3T3 Fibroblasts* *Transfected with EGFP-actin*	*Custom-built device* *Silicon membranes* *Fibronectin coating*	*6 to 32% in 2 s and kept stretched for 10 m, relaxation membrane* *within 2 s*	*SFs disassembly during stretching* *New filament bundling occurs after stretch*	*Wang* *et al.* 150
*REF-52 Rat Embryonic Fibroblasts* *Transfected with Life Act*	*Custom-built device* *Silicon membranes* *Fibronectin coating* 70	*8% at 4 Hz for 90 m*	*Cell reorient by realigning pre-existing SFs* *GFP-actin fusion proteins influence the mechanical behavior of cells*	*Deibler* *et al.* 36
*NIH3T3 Fibroblasts* *Transfected with GFP-LifeAct and mCherry-Vinculin*		*8% at 0.1 to 3 Hz*	*Increasing frequency induces less spreading* *Above 1 Hz level of perpendicular cell reorientation is not further increased* *Disruption of contractility affects cells reorientation* *SFs rather form de novo in the perpendicular direction where low mechanical forces are acting on the cell*	*Greiner* *et al.* 51
*REF-52 Fibroblasts* *Stably expressing YFP-paxillin*		*4 to 24% at 1.2 Hz wide range of stretch configurations*	*Cell and SF orientation deviate from the zero strain and zero stress prediction*	*Livne* *et al.* 89
U2OS Osteosarcoma cells Stably expressing GFP-Actin	STREX Silicon chambers Fibronectin coating	Different waves for 10.5 h	Reorientation depends on strain rate	Tondon *et al.* 133
Rat Bone Marrow Mesenchymal Stem Cells (BMSCs)	Custom-built device Silicon membranes Gelatine coating	10% at 1 Hz for 0 to 36 h	Cell reorientation depends on strain duration Cell reorganization depends on the duration of the stretching	Zhang *et al.* 165

Live imaging and study of SFs reorientation dynamics are emphasized in italics

*SF* stress fiber, *FA* focal adhesion, *PDMS* polydimethylsiloxane, *JNK* c-Jun N-terminal kinase

Most of these studies are carried out with custom-built devices for which an accurate and rigorous strain characterization is needed, but often overlooked. These devices are made of motorized stages capable to stretch silicone membranes coated with extracellular matrix proteins such as fibronectin or collagen. Given the mechanical properties of the elastomeric materials, once the membrane is stretched along one direction, it contracts in the perpendicular direction (Poisson’s effect). New commercially available devices have been designed to avoid this drawback (FlexCell^5^,^88^,^126^ and STREX^133^). Nevertheless, the use of such diverse instrumentations cannot help to distinguish between the impacts of the different factors. Moreover, the interference of signaling mechanisms cannot be excluded when different coatings are employed. Altogether, controlled experimental conditions are needed towards a comprehensive understanding of cell reorientation.

Efforts to unravel the spatiotemporal dynamics of cellular adaptations especially at the level of stress fibers are still limited. Most of the observations come from a state-to-state like manner, making use of fixed cells that do not allow observations of subcellular dynamics. The technological challenges that must be overcome include the use of actin stress fiber probes that do not interfere with the dynamics of actin polymerization and the mechanical properties.^36^ Moreover, the timescale of actin reorganization pushes further the experimental limits.

From time-lapse studies, it has been established that cells become nearly round in the first phases of reorientation and, subsequently elongate along the strain avoidance direction.^59^,^70^,^101^ During this second phase, a process of reinforcement and repair of the stress fiber strain sites occurs. Zyxin is recruited at strain-induced damage sites of stress fibers and subsequently activates actin cytoskeleton repair and reinforcement.^55^,^84^,^123^,^124^,^162^ In terms of temporal dynamics, stress fibers significantly anticipate cell overall reorientation. Stress fiber reorganization response occurs within the first minutes from the onset of the cyclic strain stimulation, while complete cell reorientation is seen in the time range of hours.^32^,^59^ In 2001, Hayakawa *et al.* observed in rat smooth muscle cells the breakdown of stress fibers aligned along the stretching direction soon after the start of the mechanical stimulation, followed by stress fiber reorientation at an oblique angle with respect to the axis of stretching.^60^ Similar observations were reported by Ngu *et al.* in bovine endothelial aortic cells.^101^ Also, the investigation of Lee *et al.* pointed out that bovine aortic endothelial cell reorientation involved the disassembly of the stress fiber proximal section (far from the focal adhesions) and de-novo formation of stress fiber at a reoriented angle with comparatively little focal adhesion turnover.^82^ These studies suggest that reorientation of stress fiber takes place through stress fiber turnover and re-assembly. However, there is also another line of evidence which suggests that stress fiber turnover might occur *via* focal adhesion sliding and consequent stress fiber rotation. Deibler *et al.*^36^ demonstrated that rat embryonic fibroblasts reorient by realigning pre-existing stress fiber while Goldyn *et al.*^48^ tracked the dramatical sliding of focal adhesions induced by uniaxial cyclic strain in NIH3T3 fibroblasts. Most probably, the aforementioned mechanisms are not mutually exclusive. Still, the challenge for the future remains to uncover the precise mechanisms of stress fiber and cell reorientation, by focusing on the heterogeneity observed not only on subcellular locations but also along the same stress fibers.^1^,^82^

A number of theoretical models have been elaborated in the endeavor to describe the relationship between the actin cytoskeleton reorganization and the uniaxial cyclic strain acting on cells. In 2000, Wang *et al.* proposed that stress fibers tend to orient in the direction of minimal normal strain, where the unperturbed state is maintained.^143^ Other models, mostly based on the molecular aspects of stress fiber assembly,^72^,^106^,^152^ were developed based on the same approach. Instead, the work of De *et al.* predicts stress fiber orientation in the minimal matrix stress direction using a coarse-grained model of cells approximated as single force dipoles.^34^ While consistency between the predictions of these models and experimental results was proven in many studies, recently, Livne *et al.* have found significant deviation between their results and the theoretical predictions proposed by the existing models.^89^ By investigating strain avoidance response over a wide range of stretch configurations, they demonstrated that stress fiber reorganization does not coincide with the direction of minimal strain or stress of the substrate. Therefore, they developed a new theoretical approach based on the molecular and physical properties of the stress fiber-focal adhesion system. Yet, it remains to be tested whether this model is cell-type independent.

### Cellular Orientation Response to Combined Substrate Anisotropy and Uniaxial Cyclic Strain

From the previous paragraphs it appears that cell and stress fiber orientation can be influenced by anisotropic cues or by imposing uniaxial cyclic strain on cell growth substrates. This legitimates to ask what the influence of anisotropic cues and cyclic strain is when these cues are applied in combination and along the same direction. This simultaneous stimulation, theoretically, would lead to competing stimuli for cell reorientation. An overview of the studies conducted applying anisotropic cues and uniaxial cyclic strain are reported in Table 3. We have considered all cell types, as the number of these studies is limited.

**Table 3:** Experimental studies about cell and stress fiber orientation response to combined topography and uniaxial cyclic strain

Cell type	Anisotropic cues	Stretching method and parameters	Main results	Source
MC3T3-E1 osteoblasts	Parallel microgrooves: 1.6 *μ*m depth and varying groove/ridge widths from 1 to 6 *μ*m ProNectin-F coating Parallel to stretching direction	Custom-built device^148^ Silicon membranes 4% at 1 Hz for 20 days	Cells align with grooves independently of topographic features SFs highly aligned and elongated after stretch.	Wang *et al.* 147
Human Skin Fibroblasts	Parallel microgrooves: 1.6 *μ*m depth, with widths from 1 to 6 *μ*m (2 to 6 *μ*m wide ridge) ProNectin-F coating Parallel to stretching direction	Custom-built device^148^ Silicon membranes 4 to 12% at 1 Hz for 24 days	Until 8% stretch, cells maintain the alignment imposed by the microgrooves, regardless of their dimensions For high strain (12%) and small microgrooves (1 *μ*m width and 2 ridge width) cells change orientations Dimension of the microgrooves and the strain magnitude are two important factors in determining cell alignment	Wang *et al.* 146
Human Patellar Tendon Fibroblasts	Parallel microgrooves: 3 *μ*m depth and 10 *μ*m width ProNectin-F coating Parallel to stretching direction	Custom-built device^148^ Silicon membranes 8% at 0.5 Hz for 72 h	Cells do not change alignment, regardless of the alignment to stretching direction	Wang *et al.* 151
Mesenchymal Stem Cells	Parallel microgrooves: 10 *μ*m width and 3 *μ*m height Parallel and perpendicular to strain direction	Custom-built device Silicone membranes Rat tail collagen I coating 5% at 1 Hz for 2–4 days	Cells and SFs remain well aligned with the microgrooves in both parallel and perpendicular microgrooves Distinct effects on gene expression in parallel and perpendicular microgrooves	Kurpinski *et al.* 79
Bovine Vascular Smooth Muscle Cells	Parallel microgrooves of varying widths Human plasma fibronectin coating Parallel or perpendicular to the direction of strain	Custom-built device Elastomeric membranes 10% at 1 Hz for 24 h	Strain parallel to microgrooves limit cell orientation response, small (15 *μ*m) and large (70 *μ*m) grooves are more favorable for reorientation than average size (40 *μ*m) Strain perpendicular to microgrooves: enhanced cellular alignment	Houtchens *et al.* 65
C2C12 Skeletal Myoblasts	Parallel microcontact printed fibronectin lines: 30 *μ*m width, 40 um spacing on hydrogels Arranged parallel, horizontal and 45° to strain direction	Custom-built device^70^ Elastomeric membranes 7% at 0.5 Hz for 4 days	SFs reorient while cell are geometrically constrained to the lines Parallel lines: SFs reorient at 48°, with a large scatter Perpendicular lines: SFs reorient at 91° Lines at 45°: SFs reorient at 52°	Ahmed *et al.* 2
Rat Bone Marrow Mesenchymal Stem Cells	Parallel nano- and micro-grooves: 300 nm width and 60 nm depth (600 nm pitch) and 1 *μ*m wide, and 500 nm deep (pitch 2 *μ*m) RFGD treatment Parallel to strain direction	Custom-built device^100^ Elastomeric membranes 1 to 8% at 1 Hz with intermittent stretch duration (15 min stretch/15 min rest for 16 h, followed by 8 h of rest)	Nanodimensions induce less alignment than microdimensions in static conditions Cells have strain avoidance response on nanotextured surface but not on micrometer-sized textures	Prodanov *et al.* 112
Human Vena Saphena Cells (Myofibroblasts)	Elastomeric microposts Elliptical cross-section (major semi-axis minor semi-axis) 1, 3 and 6 *μ*m height Human Plasma Fibronectin microcontact printed coating on top of microposts	FlexCell 7% at 0.5 Hz for 19 h	Competition between contact guidance and strain avoidance results from distinct behavior of actin cap and basal actin layer	Tamiello *et al.* 128

*SF* stress fiber, *RFGD* radio frequency glow discharge

By using microgrooves integrated in a custom-built stretching device, Wang and Grood demonstrated that micro-topography generally overrules strain avoidance, i.e., adherent cells maintain the original orientation imposed by the microgrooves, even if strain stimulation occurs along the same direction.^146^,^147^,^151^ Prodanov *et al.* have added to this evidence that cellular response might be influenced by the dimension of the anisotropic textures.^112^ They showed that osteoblasts plated on nanogrooves and subjected to cyclic strain responded by strain avoidance, while on micro-sized features, they remained aligned with the anisotropy of the substrate. Next to this, it has been observed by Ahmed *et al.* that topographical cues combined with cyclic strain can have distinct impact on stress fibers as compared to cell body reorientation response.^2^ In their study, myoblasts were confined on substrates patterned with parallel fibronectin lines (widths comparable to cell size) and exposed to uniaxial cyclic stretch. It appeared that, while cell bodies remained confined on the micropatterned lines, stress fiber succeeded in reorganizing perpendicular to the strain direction. This points to different mechanisms involved in strain and anisotropy sensing.

**Figure 5:**
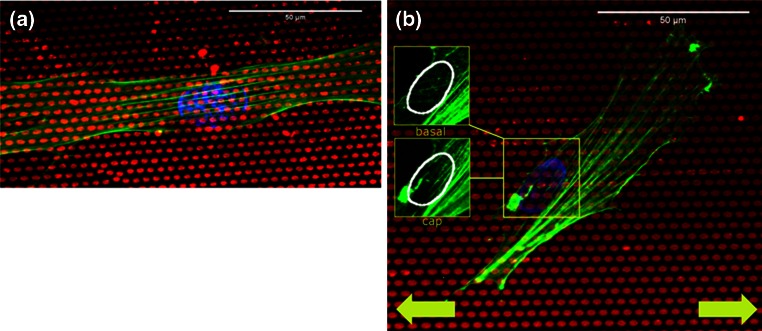
Cellular orientation response to combined substrate anisotropy and uniaxial cyclic strain. (a) Myofibroblasts (Human Vena Saphena Cells) cultured on top of fibronectin-coated elliptical microposts (red) in static conditions for 6 h. The stress fibers, colored in green, orient along the substrate anisotropy, i.e., along the micropost major axis. The nucleus is shown in blue. (b) The system made of elliptical microposts can be stretched along the micropost major axis (horizontal direction, yellow arrows). The use of this model system revealed that, the orientation response of myofibroblasts exposed to substrate anisotropy and strain (19 h, 7%, 0.5 Hz) along the same direction is determined by the distinct response of the actin stress fibers running on top of the nucleus (inset cap) and the ones present underneath the nucleus and connected to the microposts (inset basal). While the cap actin fibers respond by strain avoidance, the basal stress fibers tend to follow the direction on the micropost major axis.^118^

Recently, a study from our group has provided further insight on the mechanisms underlying stress fiber response to combined cyclic strain and anisotropic cues. It was observed that distinct responses occur at the actin cap and basal layer (Fig. 5). The actin cap stress fibers clearly tend to neglect the topographical cues and respond to strain, while the basal actin fibers remain aligned with the topography of the substrate.^128^ These findings provided evidence that cellular response to anisotropy of the substrate and cyclic strain is the complex integration of subcellular structural responses. Nevertheless, most of the mentioned studies reported on cell and stress fiber orientation but neglected the response of crucial structures such as focal adhesions.

Altogether, the examples reported above show that, although general indication exists that anisotropic cues modulate cell and stress fiber orientation response to uniaxial cyclic strain, a deeper understanding of the phenomenon is still needed. Detailed quantification of stress fiber reorientation dynamics at the subcellular level upon presentation of simultaneous anisotropic and cyclic strain cues would be of great benefit for unraveling the temporal dynamics of the processes involved in cellular adaptation.

## Summary and Outlook

A deep understanding of the mechanisms by which biophysical cues regulate cellular orientation is fundamental for cardiovascular regeneration strategies, such as biomaterial-based *in situ* engineering approaches that need to guide and control cell and tissue organization for proper tissue functioning. In particular, cell alignment is a primary aim of regeneration of cardiovascular tissues, as controlled cellular organization is essential for matching native tissue micro architecture and functionality.^110^

In this work, we provide an overview of the knowledge obtained in the last decades about the components of the structural mechanotransduction pathway, an interconnected chain of proteins implicated in force propagation from the extracellular environment to downstream targets such as the nucleus and gene expression regulation. We also report on the current understanding of cellular orientation responses induced by the application of anisotropic cues and uniaxial cyclic strain focusing on the experimental evidence obtained with *in vitro* studies using single cell observations. The development of *in vivo*-like micro-devices has enabled researchers to perform experimental studies under controlled conditions, in the effort to uncover the link between applied biophysical cues and cellular response. Still, the large body of knowledge generated by using such diverse approaches and the cell-type dependence of the results complicate the attempt of unifying the knowledge.

All in all, the above examples demonstrate that an intact structural mechanotransduction pathway plays a crucial role in the control of normal cellular functionality. Nevertheless, in order to move forward towards understanding the complex interplay between cellular mechanoresponses and biophysical properties of the micro-environment, it is important to identify the main scientific challenges.

Firstly, further research is required to achieve an in-depth understanding of the role of the structural mechanotransduction pathway. It is necessary to determine the completeness of our current understanding of such a pathway. More importantly, it is crucial to identify the relevance of individual and combined components of this pathway for controlling cell orientation. An interesting strategy within consists in considering cells with defected structural connections or knock-out cellular models as tools for novel investigations. A first attempt has been conducted by Tamiello *et al.* (unpublished) by using actin cap-lacking fibroblasts. In this study the relevance of the actin cap in the response to anisotropic cues and strain stimulation was studied by exposing the cells to both cues, applied separately and in combination. Interestingly, since these knock-out cells have been obtained by elimination A-type lamins, they represent also a useful model for studying the development of the family of mechanotransduction diseases named laminopathies. In general, mechanotransduction studies on cells from diseased patients will not only advance our understanding of the relevance of the structural connection in the cellular mechanoresponse, but also elucidate whether diseases/disorders of mechanotransduction primarily result from structural defects, impaired biochemical signaling or a synergy between the two mechanotransduction processes. Progress in this field will eventually lead to the design of effective regenerative strategies for a variety of diseases arising for mechanotransduction defects.^69^

Secondly, future studies should focus on designing a unified systematic approach for studying cellular responses to individual biophysical cues. This is needed in order to get quantifiable measurements of the effects of the various parameters of the micro-environment on cell and stress fiber orientation, for instance. Such a simplified approach will enable the integration of the overwhelming amount of information obtained using an array of diverse devices and, consequently, enhance our knowledge about the influence of biophysical cues on cellular alignment. Once cellular responses to individual cues are established, the next step we envision is to develop integrative approaches to study cellular response to combined cues, in a more tissue-like context. This is necessary in order to unravel whether a signaling hierarchy coming from distinct cues exists. Another suggestion is to develop high-throughput systems based on the simplified systematic approach in order to screen the effects of individual and combined biophysical cues on relevant cell outputs (orientation, force, proliferation etc.). Such a systematic approach can give reliable inputs for computational models in cell mechanics to interpret experimental observations and elucidate main governing processes of cellular mechanoresponse.

Finally, more investigations are necessary to obtain an in-depth understanding of the mechanisms underlying cell mechanoresponse. To address issues on how cells integrate and transduce physical signals, it is crucial to develop live imaging techniques to analyze the structural responses at subcellular level with higher spatial and temporal resolutions. The next big technological challenge will in the application of these tools to more complex environments, such as three-dimensional (3D) substrates. These substrates are of special interest because they mimic more closely the physiological environment of tissue cells. Recent evidences suggest that cellular behaviors in 3D environments differ from observations obtained employing two-dimensional (2D) environments.

As the intricate aspects about the cellular mechanoresponse become to be better characterized, it may become possible to open new avenues for controlling the way in which cell interact and respond with their physical micro-environment. We envision this is the way forward to effectively elaborate targeted strategies for tissue regeneration and therapeutical approaches.
